# Decoding Subjective Intensity of Nociceptive Pain from Pre-stimulus and Post-stimulus Brain Activities

**DOI:** 10.3389/fncom.2016.00032

**Published:** 2016-04-14

**Authors:** Yiheng Tu, Ao Tan, Yanru Bai, Yeung Sam Hung, Zhiguo Zhang

**Affiliations:** ^1^School of Data and Computer Science, Sun Yat-Sen UniversityGuangzhou, China; ^2^Department of Electrical and Electronic Engineering, The University of Hong KongHong Kong, China; ^3^School of Chemical and Biomedical Engineering, Nanyang Technological UniversitySingapore, Singapore

**Keywords:** pre-stimulus brain activity, EEG, fMRI, pain perception, machine learning, feature selection

## Abstract

Pain is a highly subjective experience. Self-report is the gold standard for pain assessment in clinical practice, but it may not be available or reliable in some populations. Neuroimaging data, such as electroencephalography (EEG) and functional magnetic resonance imaging (fMRI), have the potential to be used to provide physiology-based and quantitative nociceptive pain assessment tools that complements self-report. However, existing neuroimaging-based nociceptive pain assessments only rely on the information in pain-evoked brain activities, but neglect the fact that the perceived intensity of pain is also encoded by ongoing brain activities prior to painful stimulation. Here, we proposed to use machine learning algorithms to decode pain intensity from both pre-stimulus ongoing and post-stimulus evoked brain activities. Neural features that were correlated with intensity of laser-evoked nociceptive pain were extracted from high-dimensional pre- and post-stimulus EEG and fMRI activities using partial least-squares regression (PLSR). Further, we used support vector machine (SVM) to predict the intensity of pain from pain-related time-frequency EEG patterns and BOLD-fMRI patterns. Results showed that combining predictive information in pre- and post-stimulus brain activities can achieve significantly better performance in classifying high-pain and low-pain and in predicting the rating of perceived pain than only using post-stimulus brain activities. Therefore, the proposed pain prediction method holds great potential in basic research and clinical applications.

## Introduction

Pain assessment is a crucial clinical practice. Inaccurate pain assessment can lead to inadequate pain management, and even misleads diagnosis and treatment (Brown et al., 2011). As a multidimensional and highly subjective experience, pain perception is primarily measured by means of self-report [e.g., Visual Analog Scales (VAS) and Numeric Rating Scales (NRS)] in clinical applications (Cruccu et al., 2008; Haanpää et al., 2011). However, the subjectivity of self-report limits its application to people with impaired consciousness (e.g., patients in a coma, vegetative state or minimal conscious state; Schnakers and Zasler, 2007) or limited cognitive capacity (e.g., young children, the elderly, patients with cognitive impairment; Wong and Baker, 1988; Herr et al., 2004; Buffum et al., 2007), and people who are unwilling to reliably communicate the feeling of pain. In addition, self-report provides limited understandings of the underlying neurophysiological processes of pain perception, which is important for the development of targeting treatments (Wager et al., 2013). Therefore, developing a neurophysiology-based pain assessment tool is highly necessary in basic pain research and clinical applications.

Non-invasive neuroimaging techniques, such as electroencephalography (EEG) and functional magnetic resonance imaging (fMRI), enable us to readily obtain brain responses to nociceptive inputs. A variety of neural correlates of nociceptive pain [e.g., laser-evoked EEG potentials (LEPs), fMRI responses within the “pain matrix”] have been identified, allowing for neurophysiology-based pain assessments (Huang et al., 2013; Wager et al., 2013). Assessing subjective intensity of nociceptive pain perception with non-invasive neuroimaging data has gained emerging interest in recent years. For example, based on the strong correlation between the amplitudes/latencies of LEP and subjective pain perception, single-trial LEP features were used to predict pain intensity with high accuracy (Huang et al., 2013). Rapid developments of neuroimaging data analytics also lead to novel and effective algorithms for pain prediction. Machine learning, which can identify brain activation patterns corresponding to pain perception, has also been used in pain prediction due to its high sensitivity to multidimensional neuroimaging patterns (Schulz et al., 2011; Brodersen et al., 2012; Wager et al., 2013).

However, conventional neuroimaging-based pain prediction methods only make use of pain-related information encoded in brain activities evoked by nociceptive stimulation, while completely overlook ongoing brain activities prior to nociceptive stimulation. Actually, pre-stimulus ongoing brain activities contain important information that is predictive of forthcoming perception of pain, because previous studies have convincingly shown that pain perception is largely modulated by ongoing cognitive states (e.g., expectation, attention, reappraisal; Terkelsen et al., 2004; Quevedo and Coghill, 2007; Wiech et al., 2008; Tu et al., 2016). Babiloni et al. (2006) firstly revealed a strong negative correlation between pre-stimulus alpha-band EEG power and subjective pain rating. The relationship between pre-stimulus brain responses and pain perception was also observed in fMRI studies. It was reported that the variability in pain perception under identical stimuli was positively correlated with the fluctuation of baseline blood oxygenation level dependent (BOLD) signal in medial thalamus, lateral fronto-parietal network, and negatively correlated with BOLD in posterior cingulate and temporo-parietal cortices (Boly et al., 2007). The pre-stimulus functional connectivity between anterior insula cortex and brainstem was also found to negatively modulate pain perception (Ploner et al., 2010). Our recent work has introduced how the ongoing fluctuations of intrinsic cortical networks (as reflected by EEG spectrogram and BOLD-fMRI responses) determine the dynamic state of the brain and influence the pain perception (Tu et al., 2016). These findings suggested that pre- and post-stimulus brain activities provide complementary information for pain encoding: post-stimulus brain activities reflect the nociceptive information while pre-stimulus brain activities are responsible for trial-to-trial variability in baseline cognitive and emotional states.

In the present work, we hypothesize that combining the information embedded in pre- and post-stimulus brain activities can lead to a more accurate prediction of nociceptive pain perception. To validate this hypothesis, we collected EEG and fMRI data in laser-evoked pain experiments and used machine learning algorithms to decode perceived pain intensity from both pre-stimulus ongoing and post-stimulus evoked brain activities. Temporal-spectral EEG spectrogram and BOLD-fMRI magnitudes (in both pre- and post-stimulus periods) comprise two high-dimensional feature sets used for pain decoding. A popular supervised machine learning method, partial least square regression (PLSR; Hu et al., 2014), was used to reduce the dimensionality of EEG or fMRI feature sets by detecting a subset of features that are closely correlated with pain. These features form a number of pre- and post-stimulus pain-related brain patterns (temporal-spectral patterns for EEG and spatial patterns for fMRI). Support vector machine (SVM; Cortes and Vapnik, 1995) was then used to decode the intensity of pain perception from the pre- and post-stimulus EEG or fMRI brain patterns. In both EEG and fMRI datasets, the proposed pain decoding method using both pre- and post-stimulus activities achieved higher prediction performance than conventional pain decoding methods using post-stimulus information only. In addition, the predictive power of pre- and post-stimulus brain patterns for pain decoding was individually assessed and ranked, helped to build a more concise prediction model and provided an understanding of to what extent the extracted pain-related patterns contribute to pain perception.

## Materials and methods

In the present work, we proposed to decode the intensity of perceived pain from both pre- and post-stimulus brain activities (sampled by EEG or fMRI) in laser-evoked pain experiments.

### Experiments

#### EEG experiments

EEG data were collected from 96 healthy participants (51 females) aged 21.6 ± 1.7 years (mean ± *SD*). All participants gave their written informed consent and the experimental procedures were approved by the local ethics committee. Details of experimental design and recordings have been published previously (Hu et al., 2014).

In brief, nociceptive-specific radiant-heat stimuli were generated by laser and a total of 40 pulses, 10 for each of the four stimulus energies (E1: 2.5 J; E2: 3.0 J; E3, 3.5 J; E4, 4.0 J), were delivered in a pseudorandom order. The inter-stimulus interval varied between 10 and 15 s. After each stimulus, subjects were instructed to rate the intensity of the painful sensation elicited by the laser pulse, using a visual analog scale (VAS) ranging from 0 to 10 (0 corresponds to “no pain,” “<5” corresponds to “heat pain,” “≥5” corresponds to “acute pain,” and “10” corresponds to “pain as bad as it could be”; Jensen and Karoly, 1992). EEG data were continuously recorded using 64 Ag-AgCl scalp electrodes placed according to the International 10–20 system (Brain Products GmbH; Munich, Germany; pass-band: 0.01–100 Hz; sampling rate: 1000 Hz), using the nose as reference. Electrode impedances were kept below 10 kΩ. Electro-oculographic (EOG) signals were simultaneously recorded using surface electrodes to monitor ocular movements and eye blinks.

#### Functional MRI experiments

Functional MRI data were collected from 32 healthy participants (20 females) aged 22.1 ± 2.0 years (mean ± *SD*). All participants gave their written informed consent and the experimental procedures were approved by the local ethics committee. The current dataset follows a similar experimental design as was adopted in the EEG dataset, with the exception that inter-stimulus interval was longer (varied between 27 and 33 s) due to the low temporal resolution of fMRI recording. Functional MRI data were acquired using a Siemens 3.0 Tesla Trio scanner with a standard head coil. A whole-brain gradient-echo, echo-planar-imaging sequence was used for functional scanning with a repetition time (TR) of 1500 ms (29 ms echo time, 25 5.0 mm-thick slices with 0.5 mm inter-slice gaps, 3 × 3 mm in-plane resolution, field of view 192 × 192 mm, matrix 64 × 64; flip angle 90°). A high-resolution T1-weighted structural image (1 mm^3^ isotropic voxel MPRAGE) was acquired after functional imaging.

### Methods

**Figure 1:**
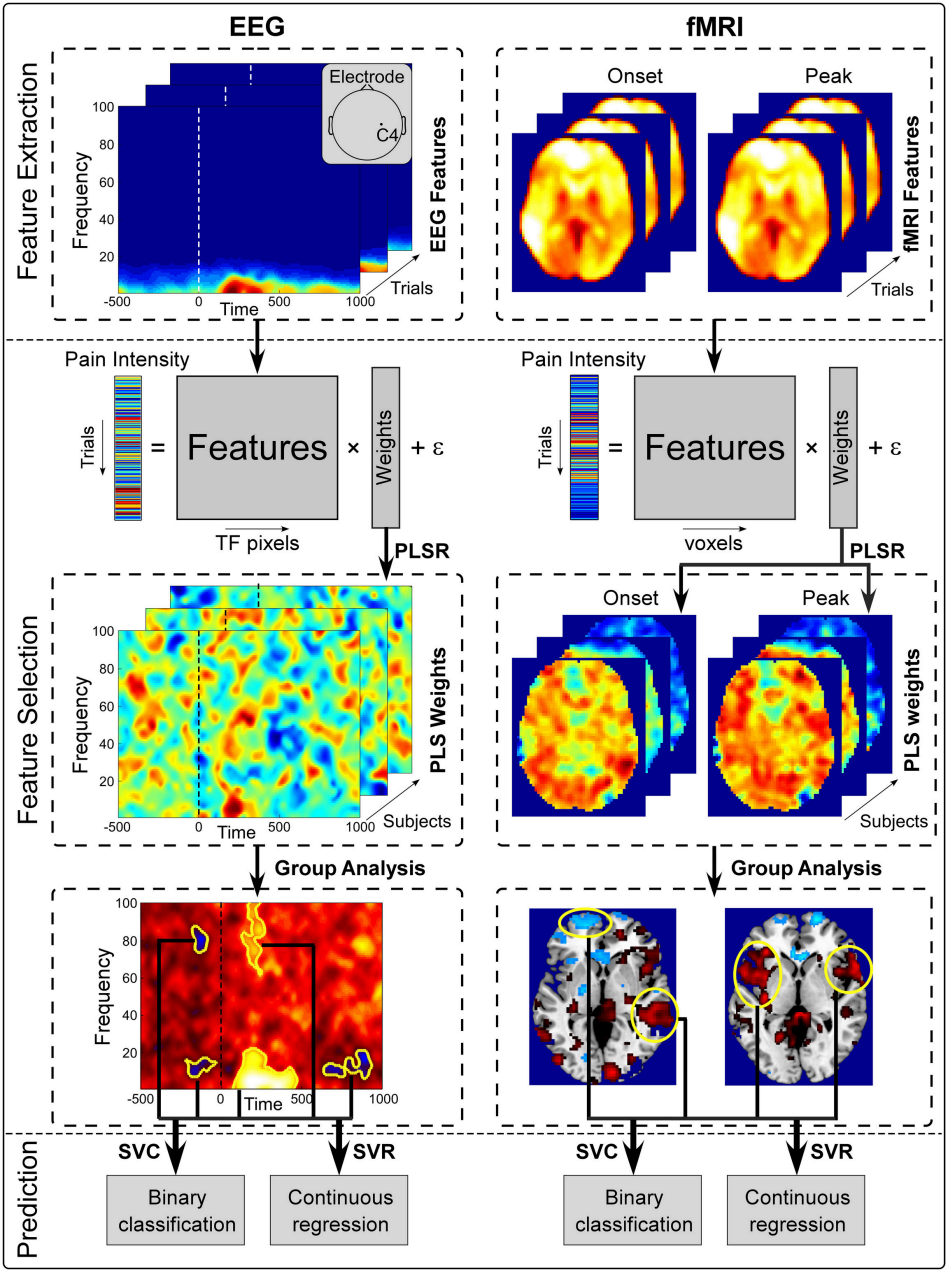
**Overview of the proposed pain decoding method (pre-processing steps are not shown)**. (1) *Feature extraction*. Single-trial pre- and post-stimulus EEG spectrogram at C4 electrode (**left**) and whole-brain fMRI BOLD signals of onset and peak scans (**right**) were extracted from preprocessed data as features. (2) *Feature selection*. Extracted features and perceived intensity of pain were fitted in a PLSR model. Features that were significantly predictive of pain perception were identified from group analysis and form a set of patterns (temporal-spectral EEG patetrns and spatial fMRI patterns). (3) *Prediction*. Selected brain patterns were fed to SVC and SVR models for pain classification and regression.

**Figure 2:**
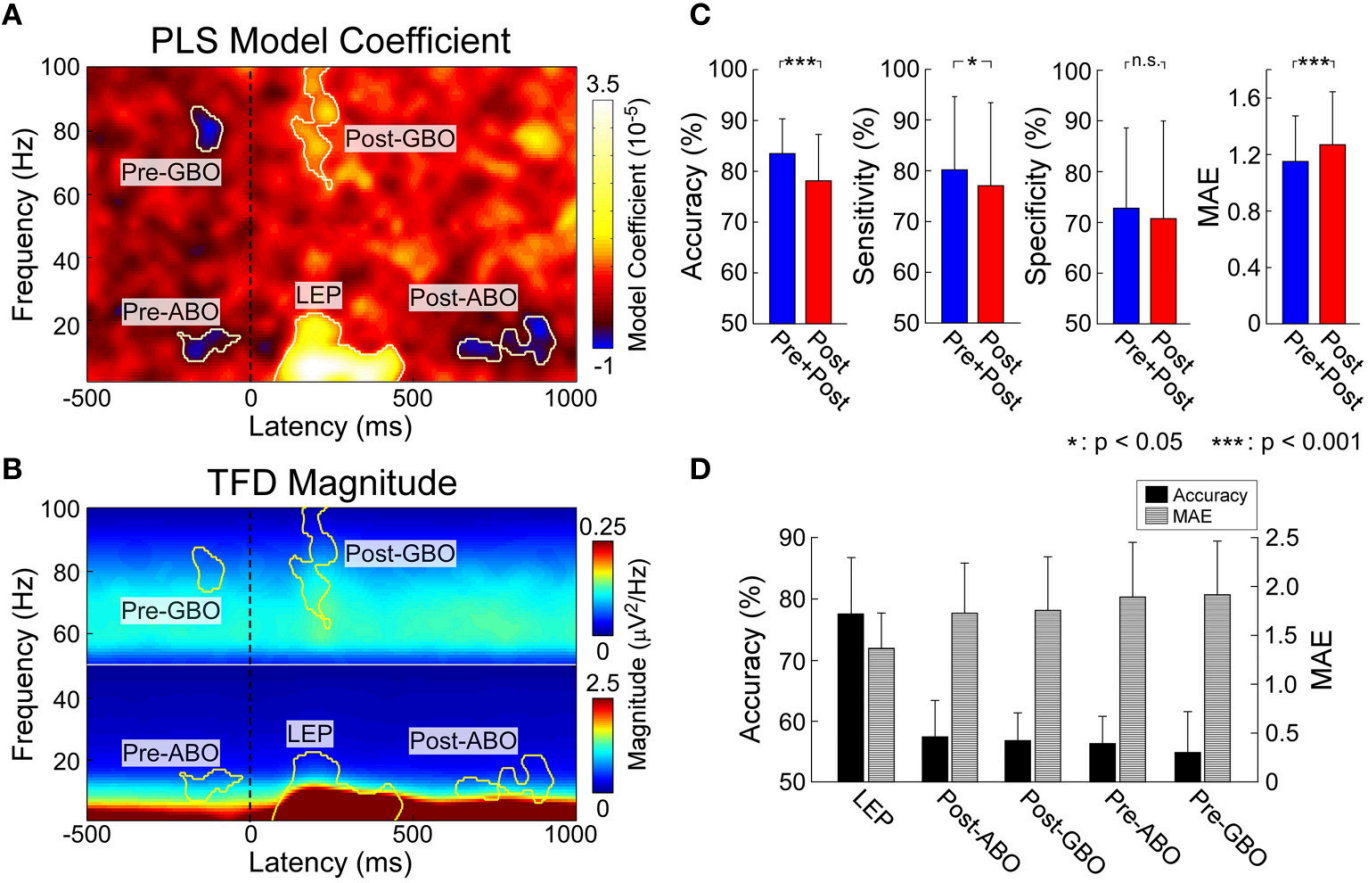
**(A)** Identified pain related EEG time-frequency patterns. PLSR coefficients indicating the relationship between EEG spectrogram and the intensity of pain. Five time-frequency clusters (C4-nose) significantly modulated the intensity of pain. **(B)** Group-level average of EEG spectrogram (C4-nose). Time-frequency power within corresponding five time-frequency clusters were selected for pain prediction. **(C)** The performance to predict subjective intensity of pain based on “Pre+Post” and “Post” EEG features. Classification accuracies to discriminate low and high pain trials were 83.5 ± 6.8% (“Pre+Post”) and 78.2 ± 9.1% (“Post”), respectively (*p* < 0.0001, paired *t*-test). Significant difference was observed in classification sensitivity (*p* < 0.05, paired *t*-test), but not in specificity. Prediction errors to continuous predict pain intensity were 1.15 ± 0.32 (“Pre+Post”) and 1.27 ± 0.38 (“Post”), respectively (*p* < 0.0001, paired *t*-test). Error bars represent *SD* across subjects. **(D)** The performance to predict subjective intensity of pain based on individual EEG patterns. “LEP” provided strongest and most significant prediction performance.

The proposed pain decoding pipeline is shown in Figure 1. The pipeline consists of 3 steps: (1) pre-processing (not shown in Figures 1, 2 feature extraction and selection; and (3) pain prediction. Firstly, pre-processing is aimed to remove noise and artifacts from raw EEG and fMRI recordings. Secondly, a subset of pain-related features is selected from high-dimensional neuroimaging data (time-frequency EEG data or whole-brain fMRI data) in both pre- and post-stimulus periods to form discriminative temporal-spectral EEG patterns and spatial fMRI patterns. Thirdly, a prediction model is established to describe the relationship between the level of pain perception and identified EEG or fMRI patterns in both pre- and post-stimulus periods. Two machine learning methods, PLSR and SVM, were used in step 2 and step 3, respectively. Although, EEG and fMRI have different pre-processing steps, they share similar methods in the steps of feature selection and prediction.

#### Pre-processing

For EEG data, five subjects were excluded from the dataset since they did not have variable painful sensation in response to different stimulus energies. EEG data were preprocessed using EEGLAB (Delorme and Makeig, 2004) and underwent standard pre-processing. Continuous data were filtered (1–100 Hz) and segmented into epochs (–500 to 0 ms and 0 to 1000 ms for pre- and post-stimulus, respectively) and baseline-corrected using pre-stimulus interval. An infomax independent component analysis (ICA; Delorme and Makeig, 2004) was used to correct trials contaminated by eye blinks and movements.

**Figure 3:**
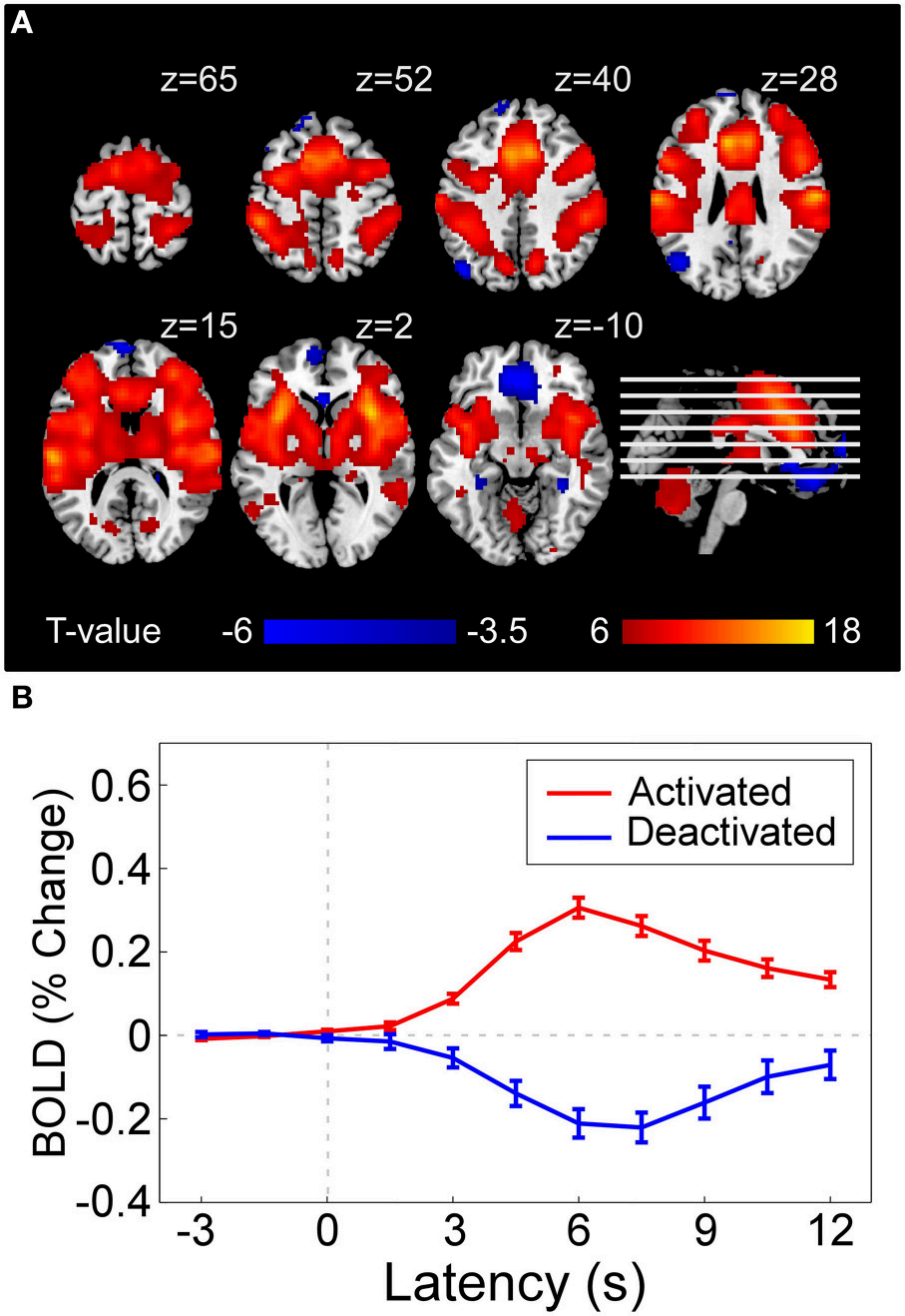
**(A)** Statistical result of brain regions activated (red) and deactivated (blue) by nociceptive pain following a conventional GLM analysis in SPM8, which represents the voxel-wise t-statistics of GLM model coefficients corresponding to the regressor denoting stimulus-evoked BOLD time-series (constructed by canonical hemodynamic functions at stimulus onsets) at group level [corrected with false discovery rate correction (FDR)]. For illustrative purpose, we used different *p*-value threshold for showing activated regions (*P*_*FDR*_ < 10^−6^, as the result was highly significant) and deactivated regions (*P*_*FDR*_ < 0.05) in the current figure. **(B)** Averaged BOLD time series (–3 to 12 s) in activated regions (red curve) and deactivated regions (blue curve). Error bar at each time instant represents the standard error of mean (SEM) of BOLD responses across subjects.

For fMRI data, two subjects were excluded from the dataset since they did not have variable painful sensation in response to different stimulus energies. The preprocessing routine was conducted using SPM8 (Wellcome Trust Center for Neuroimaging, London, UK). Images were slice-timing corrected, head motion corrected, normalized to the Montreal Neurological Institute (MNI) space (voxel size = 3 × 3 × 3 mm) by mapping T1-weighted structural image to MNI template (Ashburner and Friston, 2005), and spatially smoothed using a Gaussian kernel of 8 mm full width at half maximum (FWHM = 8 mm). A high-pass filter was applied (cut-off frequency = 1/128 Hz) to the BOLD time-series to remove low-frequency drifts. BOLD responses were modeled as a series of events using a stick function and ratings were included as a parametric modulator of each stimulus, which were then convolved with a canonical hemodynamic response function (HRF). Group-level statistical analyses were carried out using a random effects analysis with one-sample *t*-test as implemented in SPM8. Brain regions activated by laser stimuli were illustrated in Figure 3.

#### Feature extraction and selection

EEG spectral power in the time-frequency domain and BOLD-fMRI strength are used as features to predict pain levels. For EEG, short-time Fourier transform (STFT) with a fixed 200 ms Hanning window (Zhang et al., 2012) was applied to single-trial data at electrode C4 (LEP has maximal responses at the contralateral site of somatosensory area) (Valentini et al., 2012) to obtain their time-frequency distributions. Pre-stimulus (–500 to 0 ms) and post-stimulus (0 to 1000 ms) EEG spectrograms were extracted as pre- and post-stimulus features (i.e., each feature represents the power at a time-frequency pixel in the spectrogram) for further analysis. For fMRI, the whole-brain scan at stimulus onset containing pre-stimulus brain patterns immediately before stimulus onset (onset scan), and the whole-brain scan corresponding to the maximum BOLD response to nociceptive pain (peak scan, i.e., 4th scan after stimulus onset, Figure 3B), were extracted as pre- and post-stimulus features (i.e., each feature represents a voxel at stimulus onset or response peak in the scans) for further analysis. Since in both EEG and fMRI experiments painful stimuli were delivered in 4 different energy levels, both EEG and fMRI features as well as subjective pain ratings were normalized by removing the mean values of ratings within each energy group to minimize the influence of stimulus energy on the assessment of their trial-to-trial relationship.

For each subject, a linear model is used to describe the relationship between the level of pain perception and pain-related neuroimaging features, which include pre-stimulus features ($X_{m}^{pre}$, *m* = 1, …, *M*, where *m* denotes the index of pre-stimulus features and *M* is the total number of pre-stimulus features) and post-stimulus features ($X_{n}^{post}$, *n* = 1, …, *N*, where *n* denotes the index of post-stimulus features and *N* is the total number of post-stimulus features). The linear function linking the reported intensity of pain, *Y*, and EEG or fMRI features of one trial reads:

(1) $$\begin{array}{l} Y=a_{0}+\overset{M}{\underset{m}{\sum }}a_{m}^{pre}X_{m}^{pre}+\overset{N}{\underset{n}{\sum }}a_{n}^{post}X_{n}^{post}+\varepsilon , \end{array}$$

where $a_{m}^{pre}$ and $a_{n}^{post}$, respectively, denote the model coefficients for $X_{m}^{pre}$ and $X_{n}^{post}$, *a*_0_ denotes the intercept, and ε denotes the model residual.

Following, a subset of features that are most predictive of pain perception was selected. Here, the predictive power of each feature was defined according to its corresponding model coefficient ($a_{m}^{pre}$ or $a_{n}^{post}$) in Equation (1). That is, those features with a corresponding model coefficient significantly different from 0 across subjects were regarded as regions with predictive power. To achieve this, PLSR [implemented by Nonlinear Iterative Partial Least Squares algorithm (NIPALS); Wold et al., 2001] was applied to estimate the model coefficients in Equation (1). PLSR was applied here as it can solve the problems of high dimensionality and multicollinearity, which are typical in neuroimaging data. Statistical significance of the estimated model coefficients across subjects were assessed with a point-by-point one-sample *t*-test against zero, combined with nonparametric permutation testing (see PLSR analysis in Tu et al., 2016 for details of this method). The statistical result defines a number of pain-related patterns (temporal-spectral patterns for EEG and spatial patterns for fMRI), which consist of features that are most predictive of pain intensity and share similar temporal-spectral characteristics (for EEG) or spatial characteristics (for fMRI) across subjects. More precisely, for EEG data, these patterns are neighboring time-frequency pixels having power values that are significantly correlated with pain perception, while for fMRI data, these patterns are neighboring voxels having BOLD strengths that are significantly correlated with pain perception.

#### Pain prediction

In this step, pain prediction models were trained to decode single-trial intensity of pain perception from identified pain-related patterns (time-frequency patterns for EEG and spatial patterns for fMRI). Two types of pain decoding models were trained in the current study: (1) classification, which qualitatively predicts the intensity of pain by classifying trials into two levels (low pain: VAS < 5; high pain: VAS ≥ 5); and (2) regression, which quantitatively predicts the intensity of pain as a continuous value (0–10). Linear support vector classification (SVC) and support vector regression (SVR) model were, respectively, adopted as the classification and regression model (Pereira et al., 2009).

A leave-one-out-cross-validation (LOOCV) strategy was adopted to evaluate the performance of the pain decoding model (SVC and SVR) for each subject (Huang et al., 2013). For each iteration in LOOCV, one trial was selected as the test sample and fed to the SVC/SVR model trained with remaining samples, and the iterations were repeated for every trial. To quantify the performance of SVC, classification accuracy, sensitivity, and specificity were calculated. Sensitivity and specificity are defined as:

(2) $$\begin{array}{r} Sensitivity=\frac{\sum TP}{\sum TP+\sum FN},Specificity=\frac{\sum TN}{\sum TN+\sum FP}, \end{array}$$

where ∑*TP* and ∑*FN* denote the number of true positive and false negative respectively, ∑*TN* and ∑*FP* denote the number of true negative and false positive respectively. Here positive is defined as high pain trials and negative is defined as low pain trials.

To quantify the performance of SVR, we used mean absolute error (MAE), which is defined as:

(3) $$\begin{array}{l} MAE=\frac{1}{P}\overset{P}{\underset{p=1}{\sum }}|R_{p}-{\hat{R}}_{p}|, \end{array}$$

where *R*_*p*_ and ${\hat{R}}_{p}$ denote, respectively, the actual and predicted intensity of pain perception for trial *p*, and *P* is the number of trials of each subject. The above steps were repeated for each subject, and the performance measures were assessed at group level (e.g., whether SVC yielded significantly above-chance classification accuracy).

Since one of our focuses is to investigate whether the combination of pain-related information in both pre- and post-stimulus information can improve pain decoding performance, we further compared the prediction performance using two sets of patterns: (1) both pre- and post-stimulus pain-related patterns (the proposed method); (2) post-stimulus pain-related patterns only (the conventional method). Last, we also evaluated the individual contribution of each pain-related brain pattern to pain decoding, yielding a ranked contribution of these pain-related patterns.

## Results

### EEG results

#### Psychophysics

Ninety-six subjects overall had an average subjective pain intensity of 5.74 ± 1.03 (mean ± *SD*). Five subjects were excluded for the following analyses since they did not have variable sensation in response to different stimulus energies. Nociceptive-specific laser stimuli of four energies (E1–E4) elicited clear pinprick sensation in the remaining 91 subjects (E1: 3.81 ± 1.41; E2: 4.86 ± 1.34; E3: 6.63 ± 1.07; E4: 7.75 ± 1.03).

#### Pain-related time-frequency patterns

Five time-frequency clusters were identified to significantly modulate the perceived pain intensity (Figures 2A,B). In the pre-stimulus interval, a cluster in the alpha band (“Pre-ABO”: –221–31 ms, 8–15 Hz; *p* < 0.001) and a cluster in gamma band (“Pre-GBO”: –180–85 ms, 74–87 Hz; *p* = 0.001) negatively modulated the perceived intensity of a subsequent stimulus. In the post-stimulus interval, three significant clusters were observed: the low-frequency “LEP” (74–470 ms, 1–22 Hz; *p* < 0.001), the low-frequency ABO (“Post-ABO”: 637–935 ms, 8–20 Hz; *p* < 0.001), and the high-frequency GBO (“Post-GBO”: 127–377 ms, 62–100 Hz; *p* < 0.001). It was confirmed that the magnitude of “LEP” and “Post-GBO” positively correlated with perceived intensity of pain, while the magnitude of “Post-ABO” negatively correlated with perceived intensity of pain.

#### Predicting pain from pre- and post-stimulus time-frequency patterns

We trained and tested a linear SVM on two different sets of patterns: (1) post-stimulus time-frequency patterns (“Post”) including “LEP,” “Post-ABO,” and “Post-GBO”; (2) both pre- and post-stimulus time-frequency patterns (“Pre+Post”) including “Pre-ABO,” “Pre-GBO,” “LEP,” “Post-ABO,” and “Post-GBO” (Figure 2C). For classification accuracy (mean ± *SD*), “Pre+Post” provided significantly higher accuracy than “Post” (“Pre+Post”: 83.5 ± 6.8%; “Post”: 78.2 ± 9.1%; *p* < 0.0001, paired *t*-test). Significant difference was observed in classification sensitivity (“Pre+Post”: 79.2 ± 14.6%; “Post”: 77.0 ± 17.3%; *p* = 0.04, paired *t*-test), but no significant difference in specificity was observed (“Pre+Post”: 72.2 ± 14.2%; “Post”: 72.0 ± 17.3%; *p* = 0.91). For prediction error (mean ± *SD*), “Pre+Post” also provided significantly lower MAE than “Post” (“Pre+Post”: 1.15 ± 0.32; “Post”: 1.27 ± 0.38; *p* < 0.0001, paired *t*-test).

#### Predicting pain from individual time-frequency patterns

We attempted to predict the perception of pain from individual time-frequency EEG patterns, with the aim to rank their respective predictive power. Therefore, results could offer us an understanding of to what extent the extracted patterns contribute to pain prediction. It should be noticed that pre-stimulus brain patterns can only contribute to the fluctuation of perceived pain perception within identical stimuli, as the brain could not forecast the energy of forthcoming nociceptive stimuli which were randomized across trials. Therefore, we used “Pre-ABO” and “Pre-GBO” to predict the normalized intensity of pain perception, while “LEP,” “Post-ABO,” and “Post-GBO” to predict the perceived intensity of pain perception. Thus, we could propose a rank order of pain-related brain patterns (Figure 2D).

All five time-frequency patterns obtained significant prediction results. The most predictive pattern in the pre-stimulus period was “Pre-ABO” in terms of classification accuracy and prediction error (55.3 ± 6.6% and 1.79 ± 0.56) and “LEP” was the most predictive pattern in the post-stimulus period (77.0 ± 9.3% and 1.30 ± 0.36). Other patterns also provided above-chance performance (“Pre-GBO”: 55.0 ± 7.6% and 1.86 ± 0.55; “Post-ABO”: 57.5 ± 6.9% and 1.72 ± 0.51; “Post-GBO”: 56.9 ± 4.6% and 1.75 ± 0.55).

### Functional MRI results

#### Psychophysics

Thirty-two subjects overall had an average subjective pain intensity of 4.82 ± 1.55 (mean ± *SD*). Nociceptive-specific laser stimuli of four energies (E1–E4) elicited clear pinprick sensation in 30 subjects (E1: 2.92 ± 1.53; E2: 3.84 ± 1.69; E3: 5.68 ± 1.62; E4: 6.91 ± 1.54). Two subjects were excluded for the following analyses since they did not have variable sensation in response to different stimulus energies.

#### Laser-evoked BOLD responses

Single-subject fMRI data were analyzed on a voxel-by-voxel basis, using a general linear model (GLM) approach (Frackowiak et al., 2004), to assess the laser-evoked BOLD activations/deactivations. Figure 3A shows that laser stimuli elicited activations within various brain regions, including anterior/middle cingulate cortex (ACC and MCC), supplementary motor area (SMA), primary, and secondary somatosensory cortex (S1 and S2), insula (INS), and thalamus, while deactivations in rectus and DLPFC. Group-level BOLD responses in positive and negative activated regions were illustrated in Figure 3B. The peak response of positive activation was located around 6 s (4th scan) after stimulus, while was located around 7.5 s (5th scan) for deactivation.

#### Pain-related fMRI patterns

**Figure 4:**
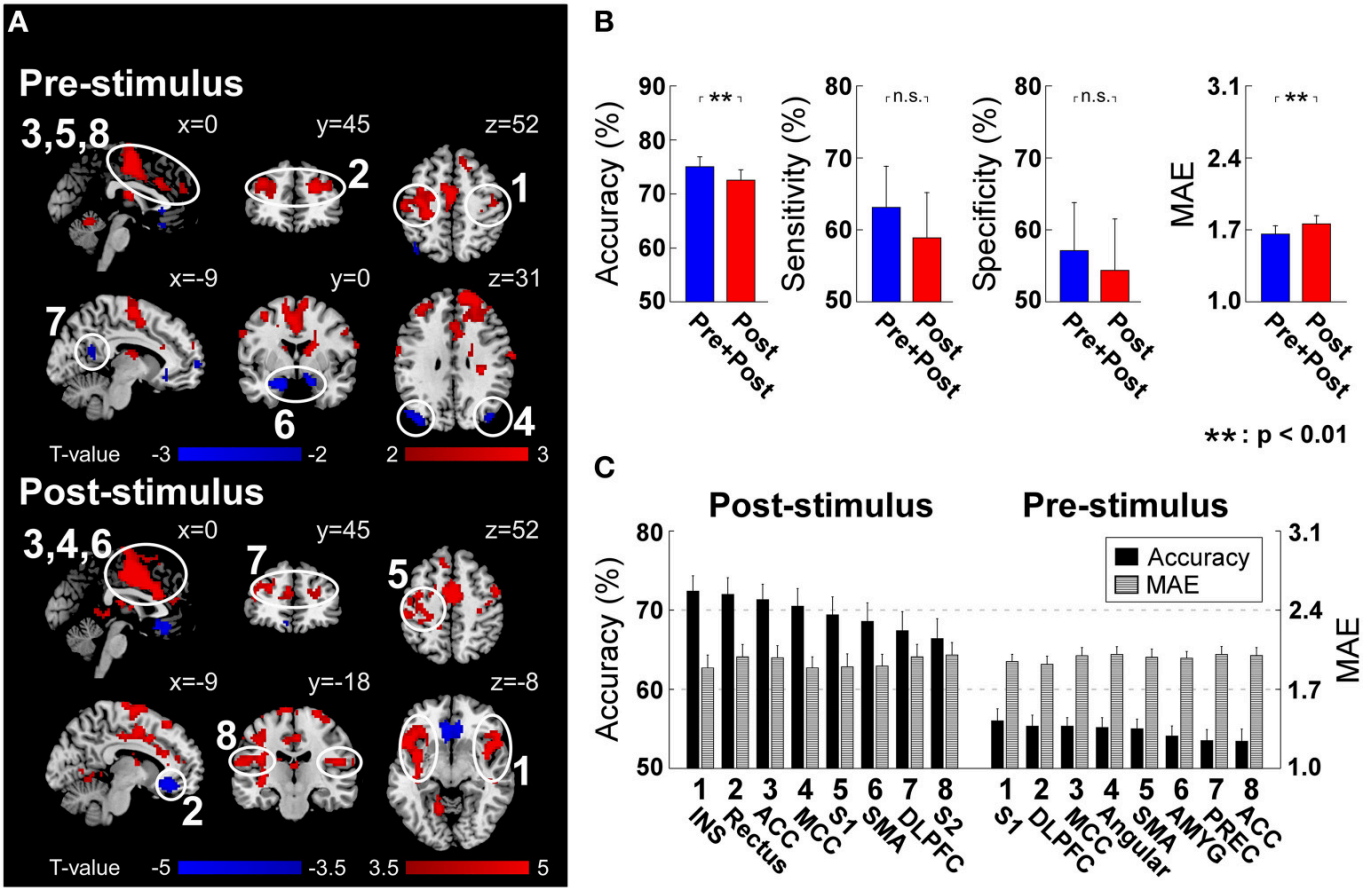
**(A)** Post-stimulus and pre-stimulus fMRI patterns defined by PLSR coefficients. Positively and negatively predictive patterns are shown in red and blue, respectively (*p* < 0.05, cluster-level permutation test). **(B)** The performance to predict subjective intensity of pain based on “Pre+Post” and “Post” fMRI patterns. Classification accuracies to discriminate low and high pain trials were 75.0 ± 10.5% (“Pre+Post”) and 72.5 ± 11.0% (“Post”), respectively (*p* = 0.0018, paired *t*-test). No significant difference was observed in classification sensitivity and specificity. Prediction errors (quantified as MAE) to continuously predict pain intensity were 1.66 ± 0.47 (“Pre+Post”) and 1.76 ± 0.47 (“Post”), respectively (*p* < 0.0035, paired *t*-test). Error bars represent SEM across subjects. **(C)** Ranked contribution of pain-related patterns. Error bars represent SEM across subjects. ACC, Anterior Cingulate Cortex; AMYG, Amygdala; DLPFC, Dorsal Lateral Prefrontal Cortex; INS, Insula; MCC, Middle Cingulate Cortex; PREC, Precuneus; SMA, Supplementary Motor Area; S1, Primary Somatosensory Cortex; S2, Secondary Somatosensory Cortex.

Post-stimulus evoked BOLD responses in several brain regions showed the capability of significantly modulating the pain perception (Figure 4A). These regions include INS, ACC, MCC, S1, SMA, and S2 in the “pain matrix” (Legrain et al., 2011), which can positively modulate pain perception, rectus in default mode network (DMN), and dorsal lateral prefrontal cortex (DLPFC), which can negatively modulate pain perception. Because of the intrinsic delay of the hemodynamic response, the fMRI signal sampled at stimulus onset reflects the brain activity preceding the arrival of the sensory input to the nervous system. At the stimulus onset time, we found a positive correlation between subsequent normalized pain intensity and BOLD in S1, DLPFC, MCC, SMA, and ACC, and a negative correlation in angular, amygdala and precuneus (Figure 4A).

#### Predicting pain from pre- and post-stimulus fMRI patterns

Similar to EEG analysis, we trained and tested linear SVM on two sets of patterns: (1) post-stimulus fMRI patterns (“Post”) including identified patterns at the peak scan; (2) both pre- and post-stimulus fMRI patterns (“Pre+Post”) including identified patterns at both onset and peak scans. For classification accuracy, “Pre+Post” provided significantly higher accuracy than “Post” (“Pre+Post”: 75.0 ± 10.5%; “Post”: 72.5 ± 11.0%; *p* = 0.0018, paired *t*-test). No significant difference was observed in classification sensitivity (“Pre+Post”: 63.1 ± 31.7%; “Post”: 58.9 ± 35.0%; *p* = 0.12, paired *t*-test) and specificity (“Pre+Post”: 57.1 ± 37.0%; “Post”: 54.4 ± 39.5%; *p* = 0.24, paired *t*-test). For prediction error, “Pre+Post” also provided significantly lower error than “Post” (“Pre+Post”: 1.66 ± 0.47; “Post”: 1.76 ± 0.47; *p* < 0.0035, paired *t*-test) (Figure 4B).

#### Predicting pain from individual fMRI patterns

We further predicted the perception of pain from individual fMRI spatial patterns. Similarly, pre-stimulus fMRI patterns were assessed to predict the normalized intensity of pain while post-stimulus fMRI patterns were assessed to predict the perceived intensity of pain (please refer to “*Predicting Pain from Individual Time-frequency Patterns*” for the reason). All regions achieved significant above-chance prediction accuracy (*p* < 0.05). The most predictive pre-stimulus fMRI patterns were S1 (56.0 ± 8.4% and 1.95 ± 0.34) (in terms of accuracy) and DLPFC (55.3 ± 7.7% and 1.92 ± 0.39) (in terms of prediction error), while the most predictive post-stimulus fMRI pattern was insula (72.4 ± 10.6% and MAE: 1.89 ± 0.62) (Figure 4C).

## Discussion

In the present work, we proposed a novel pain decoding method which uses both post-stimulus evoked brain activity and pre-stimulus brain activity as features to enhance the prediction performance compared to conventional methods based on post-stimulus evoked brain activities only. Our analysis led to two main findings.

First and foremost, our results demonstrated that by further incorporating pain-related information in pre-stimulus brain activities into the conventional pain prediction model solely based on post-stimulus evoked brain activities, the prediction performance can be significantly improved. The present work highlights the significance of pre-stimulus brain activities in encoding pain perception in the brain, and indicates the bias between actual pain perception and predicted pain perception may also be contributed from pre-stimulus brain activities.

Second, the individual predictive power of pain-related neural features is investigated and ranked, which offers us a better understanding of the predictive capacity of pain-associated brain patterns. The combined predictive power of these neural features is also obtained. Although, most of identified neural features provided above-chance prediction individually, they could not be able to yield higher predictive power when being used with other features, which implies that these regions may not provide completely independent and complimentary pain-related information.

### Significance of pre-stimulus brain activities in pain decoding

Conventional pain prediction approaches only rely on the relationship between post-stimulus evoked brain activity and pain perception, but they seldom consider the predictive power of pre-stimulus ongoing activities, which have been shown to be correlated with pain (Brodersen et al., 2012; Huang et al., 2013; Wager et al., 2013). In the present work, we demonstrated that, a prediction model, which describes the joint contribution of post-stimulus evoked brain activities and pre-stimulus ongoing brain activities to pain, can provide significantly higher prediction performance.

Actually, pre-stimulus brain oscillations have been repeatedly shown to be predictive of forthcoming sensory perception and they play an important role in the brain mechanisms underlying perception (Linkenkaer-Hansen et al., 2004; Hanslmayr et al., 2007; Van Dijk et al., 2008; Zhang and Ding, 2010; Lange et al., 2012; De Lange et al., 2013). It has been reported that the fluctuation of ongoing brain activities is able to capture the ongoing brain state and reflect various cognitive terms such as vigilance, attention, and expectation (Buzsaki, 2009). Such ongoing variation in brain state, as captured by ongoing brain activities, has been shown to be able to bias various sensory perceptions. As for pain perception, literature has shown that pain does not only reflect the neural processing of nociceptive information, but is also influenced by various psychosocial contexts and psycho-physiological factors (i.e., brain states; Wiech et al., 2008). In our previous work (Tu et al., 2016), we reported that pre-stimulus alpha and gamma oscillations sampled by EEG and BOLD activities in sensorimotor resting state network and DMN were implicated in top-down modulation of pain, and consequently modulated the perception of subsequent painful stimuli. These findings advanced our understanding of the neural mechanisms of pain, and inspired us to further utilize pre-stimulus information for pain decoding.

### Predictive neural patterns

For EEG data, when decoding pain perception from pre-stimulus activity, both “Pre-ABO” and “Pre-GBO” afforded significant accuracies. “Pre-ABO” has been interpreted as a measure of altered excitability of neuronal ensembles in primary sensory cortex, while “Pre-GBO” modulates long-range communication between distributed neuronal assembles. Thereby they offered complementary information in terms of classification accuracy. When decoding pain-related information embedded in post-stimulus EEG activities, “LEP” had the highest accuracies, indicating its strongest contribution to pain prediction [in terms of mean accuracy (%) and variability (*t*-value)]. Not surprisingly, no significant difference of prediction accuracy was observed between “LEP” and “Post” (“LEP”: 77.0 ± 9.3% and 1.30 ± 0.36; “Post”: 78.2 ± 9.1% and 1.27 ± 0.38; *p* = 0.72 and *p* = 0.54 for classification and regression, respectively). Although “Post-ABO” and “Post-GBO” enabled significant accuracies when predicting pain perception individually, they did not provide additional information when being considered along with “LEP.” It may due to the predictive information provided by “Post-ABO” and “Post-GBO” is also contained in “LEP.” Therefore, it is possible to remove “Post-ABO” and “Post-GBO” and develop a more concise pain prediction model in clinical practice.

For fMRI data, we found the activities at DLPFC are predictive of pain perception, no matter whether they are measured before or after stimulus onset. But the degrees of importance of pre-stimulus DLPFC activities and post-stimulus DLPFC activities are largely different. Pre-stimulus DLPFC activities provide the highest prediction performance among all pre-stimulus patterns (measured by MAE), showing DLPFC is one of the most important regions executing cognitive pain modulation (Wiech et al., 2008). One the other hand, post-stimulus DLPFC activities cannot offer as high prediction performance as “pain matrix” (insula, ACC, MCC, S1, and SMA) does (Legrain et al., 2011), indicating that cognitive modulation is less important after stimulus.

### Machine learning classifiers for brain decoding

Machine learning has gained popularity in the community of brain science and engineering recently for it allows for decoding stimuli, mental states, behaviors, and other variables of interest from neuroimaging data (Pereira et al., 2009). Various machine learning classifiers have been applied to brain decoding, including Logistic Regression (LR; Ryali et al., 2010), linear SVM (Ryali et al., 2010), Gaussian Naïve Bayes (GNB; Huang et al., 2013) and Fisher's Linear Discriminant Analysis (LDA) (Davatzikos et al., 2005). In the present work, we used PLSR to select discriminative features from EEG and fMRI data and applied linear SVC and SVR, both of which are extensions of the classical SVM, to classify and continuously predict subjective pain perception from EEG and fMRI features. PLSR and SVM are both popular machine learning methods and they are gradually used in many applications of brain decoding.

Since there are generally more predictors than experimental trials or subjects, it is often advantageous to reduce the number of predictors by selecting an informative subset. Wager and his colleagues used LASSO-PCR, which is combination of two dimension reduction methods (LASSO and PCA), to extract features and predict pain perception (Wager et al., 2013). LASSO is based on sparsity-enhancing *L*_1_ regularization on regression coefficients and it can shrink small regression coefficients to zero to realize dimension reduction. But, when dealing with strongly correlated predictors (e.g., adjacent fMRI voxels), LASSO arbitrarily selects one variable from a group of highly-correlated variables, which degrades the interpretability of the prediction model (Cecchi et al., 2012). Therefore, in (Wager et al., 2013), the authors first used PCA to reduce the number of predictors and then used LASSO on the orthogonal principle components (PCs) rather than the original fMRI data. However, since PCA is an unsupervised method and the PCs are obtained according to the variance of the data solely, it cannot guarantee that the classes can be well-separated in the space defined by reduced dimensions. Here we used PLSR to decode pain related brain patterns, because it is a supervised dimension reduction method that can exploit class information to ensure that high-dimensional data can be amped into a low-dimensional space where different classes are well-separated. PLSR is still a linear method but the relationship between pain intensity and brain signals could be non-linear (Wager et al., 2005; Loggia et al., 2012). There are more sophisticated methods that can explore nonlinear relationships between brain responses and behavior variables, which is in accord with intrinsic nonlinear neuro-dynamics of the brain (Tu et al., 2015).

More recently, deep machine learning algorithms which can model the data with multiple processing layers, have been applied for brain decoding and neuroscience discovery (Plis et al., 2014). What differentiates them from other classifiers is the automatic feature learning from data which largely contributes to improvements in accuracy. Deep models such as deep belief networks (DBNs) and restricted Boltzmann machine (RBM), separate linear factors from functional brain imaging data by fitting a probability distribution model to the data, has been used for fMRI classification (Schmah et al., 2008) and for identifying intrinsic networks (Hjelm et al., 2014). It is potentially a suitable solution for pain decoding model, and more advanced feature selection and machine learning techniques will be used to build a more powerful pain decoding model in our future study.

### Further developments for clinical uses

In the present study, we proposed a novel pain decoding model incorporating both pre-stimulus brain activities and post-stimulus brain activities, and adopted machine learning classifiers to effectively predict pain perception in single-trials. Such decoding model and prediction strategy could be executed rapidly, reliably, and automatically, thus satisfying most requirements of various basic and clinical applications.

Pre-stimulus brain activities could be a great indicator of subject's ongoing cognitive states and they include much useful information for decoding within-subject variability. However, our decoding model does not take inter-subject variability of pain perception and brain responses into consideration. In future, we would like to apply the decoding model on cross-subject prediction which is more favored because it does not need any training on new individuals.

EEG and fMRI are the commonly used techniques for pain assessment in clinical applications. Particularly, EEG is more favored because it is cheap and easy to operate. For pain-related clinical study, our proposed novel pain-related brain patterns hold great potential to help diagnose nociceptive system deficit as well as to predict subjective pain perception (e.g., to monitor the effect of analgesic drug or the recovery of nociceptive system for non-communicative patients). Moreover, compared with our previous work on pain prediction (Huang et al., 2013), which rely on EEG data from a high-density EEG cap, we obtain desirable pain prediction accuracy from EEG at only one electrode so that the preparation period is significantly reduced, which makes the proposed EEG-based prediction method more suitable for both clinicians and patients.

## Author contributions

YT and ZZ designed the study. AT collected the data. YT and AT analyzed the data. YT, AT, YB, YSH, and ZZ discussed the results and wrote the paper.

### Conflict of interest statement

The authors declare that the research was conducted in the absence of any commercial or financial relationships that could be construed as a potential conflict of interest.
